# The full cohort of 512 patients and the nested controlled trial in 93 patients in the Pulmonary Metastasectomy in Colorectal Cancer (PulMiCC) study raise doubts about the effective size at present claimed

**DOI:** 10.1186/s13019-022-01757-2

**Published:** 2022-01-16

**Authors:** Tom Treasure, Norman R. Williams, Fergus Macbeth

**Affiliations:** 1 Clinical Operational Research Unit, University College London, London, UK; 2 Surgical and Interventional Trials Unit, University College London, London, UK; 3 Centre for Trials Research, Cardiff University, Cardiff, UK

**Keywords:** Lung metastases, Colorectal cancer, Randomised controlled trial, PulMiCC trial, Prospective cohort study

## Abstract

A comparison of the relative merits of video-assisted pulmonary metastasectomy versus thoracotomy is predicated on the assumption that removal of asymptomatic lung metastases favourably influences survival and that it does so by a large degree. Recently published but long-awaited evidence from a prospective cohort study and a randomised trial of Pulmonary Metastasectomy in Colorectal Cancer (PulMiCC) challenges that assumption.

We read with interest the report of 483 patients with suspected lung metastases of whom 251 had metastasectomy [1]. We are grateful to Dr. Markowiak and colleagues for making reference to our study: Pulmonary Metastasectomy in Colorectal Cancer (PulMiCC). They referred to the initial publication in December 2019. This clearly documents that there was a prospective study in existence, contrary to their statement. Their summary of the circumstances needs correction. After discussion with the Independent Data Monitoring Committee, we had closed the trial in January 2017 for the explicit purpose of further follow-up and analysis of the randomised controlled trial (RCT) data and the prospective cohort study within which it was nested. We published the results in 93 randomised patients in early May 2020 [2]. While randomisation “faltered” the cohort study had recruited well to a total of 512 patients. It has also been published [3].

The 25 multidisciplinary teams recruiting patients internationally selected 263 of the non-randomised patients for lung metastasectomy and 128 to not have that operation. It is no surprise that those selected for metastasectomy had better 5-year survival which was 47% compared with 22% for those turned down. There were differences in the oncological and performance characteristics between these two groups. All differences favoured the operated group. These had been appropriately used in selecting patients most likely to live longer after metastasectomy. The data are given in the table with the hazard ratios derived by the meta-analysis of Gonzalez et al. [4] (Table 1).

**Table 1:** Favourable factors for survival were better in the metastasectomy group

Patient factors	Metastasectomy	No metastasectomy	Hazard ratio
	N = 263	N = 128	
ECOG zero^a^	68%	36%	
Median %FEV1^b^	96%	87%	
Solitary metastasis^c^	65%	31%	2.04
CEA < 5 ng/ml^d^	31%	21%	1.91
No liver metastases	36%	28%	1.22
Five-year survival	47%	22%	

^a^Easter Cooperative Oncology Group 0–5 where zero is unimpaired

^b^Forced Expiratory Volume in 1st second as a percentage of predicted values based on height and sex

^c^The hazard ratio is for multiple versus solitary. Here are given the % of patients with a solitary metastasis in each group

^d^The tumour marker carcinoembryonic antigen. All hazard ratios are from the meta-analysis of Gonzalez et al. [4]

In contrast, in the RCT these and others known confounders were very well matched and there was no difference in survival at any time point (Fig. 1) [2]. At 4 years, survival appears better in the control arm and at 5 years in the metastasectomy arm but the confidence intervals preclude claiming that either is a real difference. It is not possible to exclude a small difference in the long term. But two things are clear: the assumption of zero survival without metastasectomy [5] is contradicted, and any difference is much less than is widely believed. Analysis of health utility showed no benefit from colorectal lung metastasectomy [6].

**Fig. 1:**
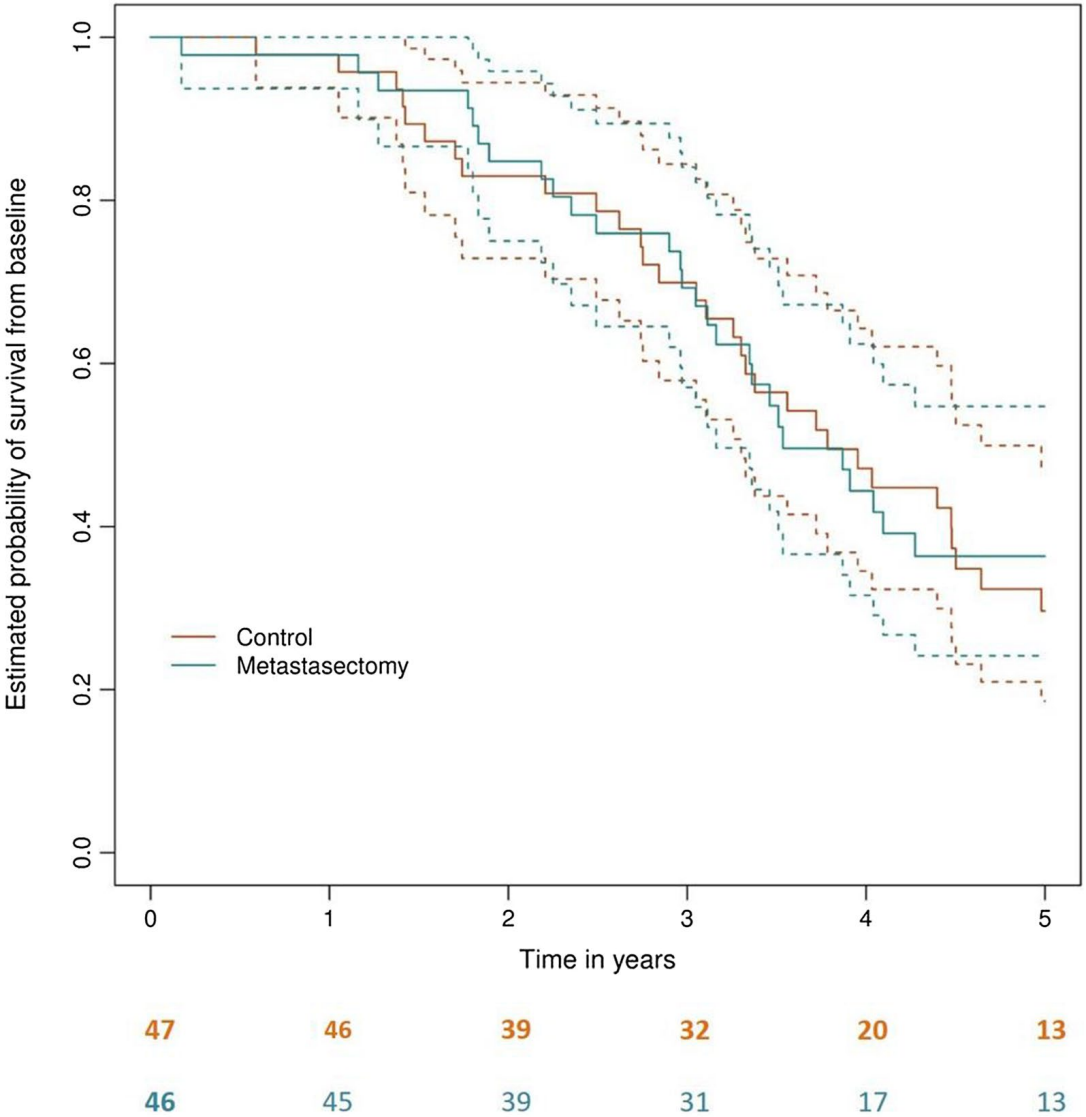
The Kaplan Meier analysis of the PulMiCC randomised controlled trial. The unadjusted hazard ratio for death within 5 years was 0.93 (95% CI 0.56–1.56). There is no significant difference at any time point with the curves weaving in and out of each other, but the median survival was longer in the control group at 3.8 (95% CI 3.1–4.6) years compared with median survival after metastasectomy 3.5 (95% CI 3.1–6.6)

The patients reported by Markowiak et al. were highly selected with high proportions of patients in both groups having a single metastasis and no extrapulmonary metastases. In the light of the PulMiCC findings it cannot be concluded that the apparently good survival in Markowiak’s study can be attributed solely, or perhaps at all, to surgery whether open or by VATS.

## Data Availability

The original data are available upon appropriate and reasonable application to the corresponding author.

## Abbreviations

RCT: Randomised controlled trial
PulMiCC: Pulmonary Metastasectomy in Colorectal Cancer
VATS: Video assisted thoracic surgery
